# Fe–Mn bimetallic oxides-catalyzed oxygen reduction reaction in alkaline direct methanol fuel cells

**DOI:** 10.1039/c7ra12610g

**Published:** 2018-02-26

**Authors:** Yuan Fang, Yonghui Wang, Fen Wang, Chengyong Shu, Jianfeng Zhu, Wenling Wu

**Affiliations:** School of Materials Science and Engineering, Shaanxi University of Science and Technology Xi'an 710021 P. R. China wangf@sust.edu.cn; State Key Laboratory for Mechanical Behavior of Materials, Xi'an Jiaotong University Xi'an 710049 P. R. China

## Abstract

Two Fe–Mn bimetallic oxides were synthesized through a facile solvothermal method without using any templates. Fe_2_O_3_/Mn_2_O_3_ is made up of Fe_2_O_3_ and Mn_2_O_3_ as confirmed *via* XRD. TEM and HRTEM observations show Fe_2_O_3_ nanoparticles uniformly dispersed on the Mn_2_O_3_ substrate and a distinct heterojunction boundary between Fe_2_O_3_ nanoparticles and Mn_2_O_3_ substrate. MnFe_2_O_4_ as a pure phase sample was also prepared and investigated in this study. The current densities in CV tests were normalized to their corresponding surface area to exclude the effect of their specific surface area. Direct methanol fuel cells (DMFCs) were equipped with bimetallic oxides as cathode catalyst, PtRu/C as the anode catalyst and PFM as the electrolyte film. CV and DMFC tests show that Fe_2_O_3_/Mn_2_O_3_(3 : 1) exhibits higher oxygen reduction reaction (ORR) activity than Fe_2_O_3_/Mn_2_O_3_(1 : 1), Fe_2_O_3_/Mn_2_O_3_(1 : 3), Fe_2_O_3_/Mn_2_O_3_(5 : 1) and MnFe_2_O_4_. The much superior catalytic performance is due to its larger surface area, the existence of numerous heterojunction interfaces and the synergistic effect between Fe_2_O_3_ and Mn_2_O_3_, which can provide numerous catalytic active sites, accelerate mass transfer, and increase ORR efficiency.

## Abstract 2

Heterojunction interfaces and synergistic effect between Fe_2_O_3_ and Mn_2_O_3_ play a key role in Fe_2_O_3_/Mn_2_O_3_-catalyzed ORR.

## Graphical abstract

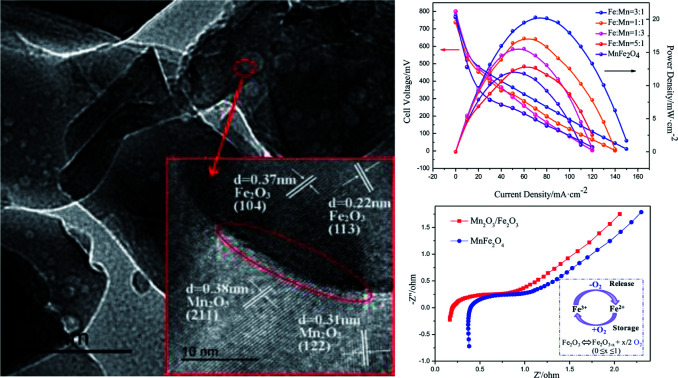

## 1. Introduction

The rapid depletion of fossil fuel and the increase in environmental pollution have driven us to search for sustainable and clean energy resources. Fuel cells have been considered promising power sources owing to their advantage of transforming chemical energy directly into electrical energy.^1^ At present, direct methanol fuel cells (DMFCs) are obtaining great attention in virtue of their high energy density, environment friendliness and comparatively lower operating temperature.^2–5^ Furthermore, methanol is convenient and safe for transport and storage, swift to refuel and available at a low price.^6^ At present, DMFCs have great potential application as a portable power supply or electric vehicle power supply. Nevertheless, the inertial oxygen reduction reaction (ORR) and methanol oxidation reaction (MOR) dynamics and the high cost of noble-based catalysts and proton exchange membrane (PEM) hinder the commercial application of DMFCs.^4,7,8^

Recently, polymer fiber membranes (PFMs) have been demonstrated to be an excellent alternative to PEMs for higher performance liquid fuel cells at a reduced cost in our previous study.^9,10^ The fibers in PFMs are neutral and possess pores and gaps, which allow molecules, ions, and liquid fuel to transport or move through the PFM freely. Consequently, the cathode catalysts should have both outstanding tolerance for methanol poisoning and excellent stability. The widely used cathode catalysts are Pt or Pt-based metal alloy catalysts, such as Pt–Co,^11^ Pt–Pd,^4^ Pt–Ni,^12^ and Pt–Fe.^13^ However, these catalysts have both ORR and MOR catalytic activity, leading to a mixed potential at the cathode and poisoning by methanol. In terms of lower cost, a variety of non-Pt catalysts, such as Ru–Se,^14^ Pd–Ni,^15^ Pd–Fe,^16^ Co–Se,^17^ Fe–N–C,^18^ Cu–Fe–S,^19^ and Co–O,^20^ which display ORR catalytic activity and better methanol tolerance than Pt-based catalysts, also have been researched.

Among them, transition metal (Fe, Co, Ni, Mn, *etc.*) oxides have gained increasing interest as ORR catalysts in virtue of their high activity, low cost and environmental friendliness.^21^ In recent years, numerous studies have focused on binary and ternary metal oxides because of their good synergistic effects and good cycle stability. NiCo_2_O_4_,^22^ KMn_8_O_16_,^23^ MnFe_2_O_4_,^24^ and Co–Ni–Te–O^25^ have higher ORR catalytic activities and methanol tolerance. For example, the catalytic activity of MnFe_2_O_4_ is higher than that of Fe_2_O_3_ (ref. 26) and Mn_2_O_3_.^27^ Nevertheless, the catalytic activity of the mixed compound of Fe_2_O_3_ and Mn_2_O_3_ has not been discussed.

In this study, we prepared two Fe–Mn bimetallic oxides, namely, Fe_2_O_3_/Mn_2_O_3_ and MnFe_2_O_4_ by a simple solvothermal method. Fe_2_O_3_/Mn_2_O_3_ is made up of Fe_2_O_3_ and Mn_2_O_3_ as confirmed *via* XRD. MnFe_2_O_4_ is a pure phase sample. The as-prepared Fe_2_O_3_/Mn_2_O_3_ exists in the form of porous nanosheets-self-assembled globular structure. The microspheres are 3–4 μm in diameter and the pore size is about 30 nm. The TEM and HRTEM images show Fe_2_O_3_ nanoparticles uniformly dispersed on the Mn_2_O_3_ substrate and a distinct heterojunction boundary between Fe_2_O_3_ nanoparticles and Mn_2_O_3_ substrate. MnFe_2_O_4_ has a hierarchical structure, in which the nanoparticles are 20–30 nm in diameter and create self-assembled globular shapes with diameters of 300–500 nm. The alkaline DMFCs were assembled using Fe_2_O_3_/Mn_2_O_3_ or MnFe_2_O_4_ as cathode catalyst, PtRu/C as anode catalyst, and PFM instead of PEM. CV and DMFC performance tests indicate that the ORR catalytic activity of Fe_2_O_3_/Mn_2_O_3_ is superior to that of MnFe_2_O_4_.

## 2. Experimental section

### 2.1 Synthesis of Fe_2_O_3_/Mn_2_O_3_ and MnFe_2_O_4_ catalysts

All reagents were analytical grade and used without further purification. All the reagents were purchased from Sinopharm Chemical Reagent Co. Ltd. The anode catalyst PtRu/C (HiSpec 3000) was bought from Johnson Matthey (UK). Multiwalled carbon nanotubes (TNM7, >95%, OD: 30–50 nm, length: 10–20 mm) were obtained from Chengdu Organic Chemicals Co. Ltd (Chengdu, China). They were produced by natural gas catalytic decomposition over a nickel-based catalyst and purified with dilute hydrochloric acid at 80 °C. The PFM (thickness 1/4 159.3 μm) was purchased from the Nippon Kodoshi Corporation.

In the synthesis of Fe_2_O_3_/Mn_2_O_3_, first, 25 mL ethylene glycol (EG) and 0.14 g Tween 80 were dissolved into 25 mL ultrapure water to form a transparent solution. Then, 3 mmol MnSO_4_·H_2_O, 9 mmol Fe(NO_3_)_3_·9H_2_O and 30 mmol urea were added to the solution, which was then magnetically stirred at 25 °C for 1 h, forming a red-brown solution. Next, the solution was put into a 100 mL Teflon-lined stainless-steel autoclave, which was then heated at 200 °C for 24 h with continuous rotation. The precipitate was washed by centrifugation with anhydrous ethanol and ultrapure water several times until the pH was 7 and the precursor of Fe_2_O_3_/Mn_2_O_3_ was obtained by drying it at 80 °C for 12 h. The resultant product was calcined at 800 °C in air for 5 h in a muffle furnace to obtain the Fe_2_O_3_/Mn_2_O_3_ sample. Fe_2_O_3_/Mn_2_O_3_ with different Fe/Mn ratios of 1 : 1, 1 : 3, 3 : 1 and 5 : 1 were prepared for comparison, which were controlled by altering the molar ratio of MnSO_4_·H_2_O and Fe(NO_3_)_3_·9H_2_O. The samples were designated as Fe_2_O_3_/Mn_2_O_3_(1 : 1), Fe_2_O_3_/Mn_2_O_3_(1 : 3), Fe_2_O_3_/Mn_2_O_3_(3 : 1) and Fe_2_O_3_/Mn_2_O_3_(5 : 1), respectively.

The precursor of MnFe_2_O_4_ was synthesized following the same solvothermal method except the raw materials were 2.5 mmol Mn(CH_3_COO)_2_·4H_2_O, 5.0 mmol FeCl_3_·6H_2_O, 1.0 g polyethylene glycol (PEG), 3.6 g CH_3_COONa and 40 mL ethylene glycol (EG). The MnFe_2_O_4_ catalyst sample was obtained after calcination at 500 °C in air for 4 h.

### 2.2 Materials characterization

The structures and compositions of the as-prepared Fe_2_O_3_/Mn_2_O_3_ and MnFe_2_O_4_ were characterized *via* X-ray diffraction (XRD, D/Max 2200PC, Japan) and high-resolution TEM (HRTEM). The morphological properties were characterized by field emission scanning electron microscopy (FESEM, Hitachi S-4800, Japan) and transmission electron microscopy (TEM, FEI company Tecnai G2 F20) equipped with energy-dispersive spectrometer (EDS). The Brunauer–Emmett–Teller (BET) method was carried out to determine the pore volumes, pore size and the specific surface area distribution of the samples using a surface area and porosimetry system (ASAP 2460, Micromeritics Instrument Corporation, USA). X-ray photoelectron spectroscopy (XPS) measurements (VG Thermo ESCALAB 250 spectrometer) were used to quantitatively analyze the chemical compositions of samples.

### 2.3 Electrochemical measurements

Cyclic voltammetry (CV) and electrochemical impedance spectroscopy (EIS) were measured using an electrochemical workstation (CHI 660E, Chenhua Instruments, Shanghai, China). A standard three-electrode system consisted of the catalyst-modified glassy carbon electrode as the working electrode, Hg/HgO electrode as the reference electrode and the Pt network as the counter electrode. The glassy carbon electrode was modified as follows: 4 mg catalyst, 1 mg CNTs, 0.2 mL distilled water, 0.5 mL absolute ethyl alcohol and 50 μL Nafion solution (5 wt%) were ultrasonically dispersed into a homogeneous suspension for about 1 h; then, the suspension was poured on the glassy carbon electrode surface and dried at room temperature.

### 2.4 Electrode preparation and DMFC measurements

The cathode electrode was a sandwich structure, including catalyst layer, current accumulating matrix and gas diffusion layer. The gas diffusion layer was obtained by mixing 60 wt% acetylene black and 40 wt% polytetrafluoroethylene (PTFE, 30 wt% solution) with ethanol under ultrasonication and pressing the slurry into a thin layer of 0.3–0.5 mm and then treating at 350 °C for 1 h. The catalyst layer was obtained first through mixing 24 mg catalyst, 6 mg CNTs and 6.7 mg 30 wt% PTFE solution into slurry with addition of a certain amount of absolute ethanol; the slurry was pasted on nickel foam (porosity > 95%) and then dried at 80 °C for 2 h. Finally, the cathode was obtained by pressing the catalyst layer on nickel foam and the gas diffusion layer under 2 MPa.

The anode was obtained *via* mixing PtRu/C (60 wt%) and Nafion solution (5 wt%) at a mass ratio of 1 : 1. The anode preparation process is consistent with that of the cathode without the gas diffusion layer. The loading of PtRu/C was 5 mg cm^−2^.

The cathode, PFM and anode were assembled into a fuel cell. At the cathode, the oxygen flow rate was 20 cubic centimeters per minute; the anode aqueous solution was 4 M KOH and 5 M methanol. The structure of PFM-DMFCs was introduced and described in our previous study.^9^ A battery testing system (Neware Technology Co., Ltd., Shenzhen, China) was used to measure the performance.

## 3. Results and discussion

### 3.1 Structural and morphological characterization

**Fig. 1:**
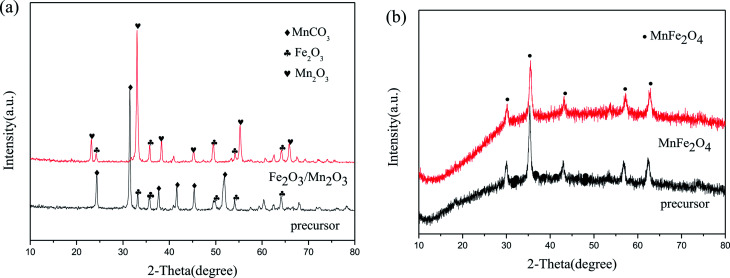
XRD patterns of (a) Fe_2_O_3_/Mn_2_O_3_(3 : 1) and its precursor; (b) MnFe_2_O_4_ and its precursor.

Fig. 1 displays the XRD patterns of Fe_2_O_3_/Mn_2_O_3_(3 : 1), MnFe_2_O_4_ and their precursors. The precursor of Fe_2_O_3_/Mn_2_O_3_(3 : 1) can be well indexed to Fe_2_O_3_ (JCPDS no. 33-0664) and MnCO_3_ (JCPDS no. 44-1472). However, the diffraction peaks of Fe_2_O_3_/Mn_2_O_3_(3 : 1) agree with the standard patterns of Fe_2_O_3_ (JCPDS no. 33-0664) and Mn_2_O_3_ (JCPDS no. 24-0508). It can be illustrated that Fe_2_O_3_/Mn_2_O_3_(3 : 1) is composed of Mn_2_O_3_ and Fe_2_O_3_ and the formation of Mn_2_O_3_ is due to the decomposition of MnCO_3_ in its precursor. Moreover, the diffraction peaks of MnFe_2_O_4_ and its precursor can be well assigned to the standard patterns of MnFe_2_O_4_ (JCPDS no. 10-0319).

**Fig. 2:**
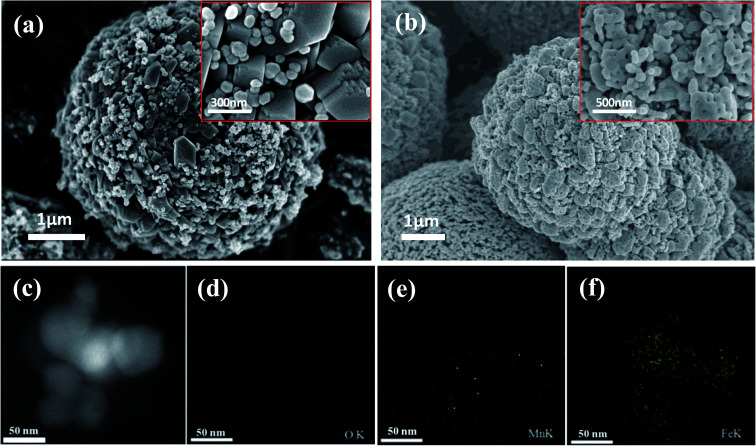
FESEM images of (a) the precursor of Fe_2_O_3_/Mn_2_O_3_(3 : 1) and (b) Fe_2_O_3_/Mn_2_O_3_(3 : 1); EDS elemental mapping images of Fe_2_O_3_/Mn_2_O_3_ ((c) to (f)).

**Fig. 3:**
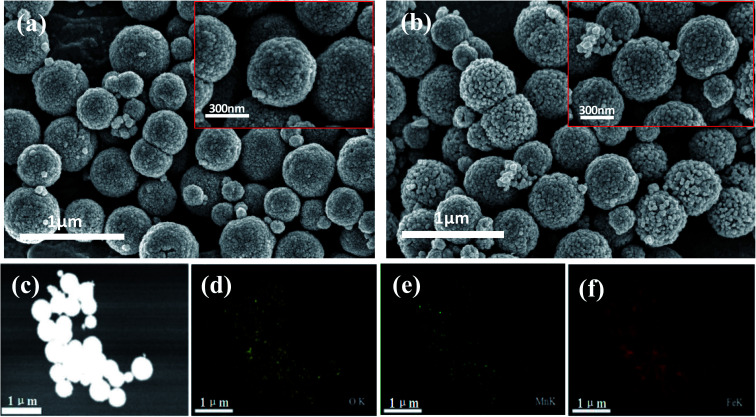
FESEM images of (a) the precursor of MnFe_2_O_4_ and (b) MnFe_2_O_4_ catalyst; EDS elemental mapping images of MnFe_2_O_4_ catalyst ((c) to (f)).

FESEM was applied to describe the morphology of Fe_2_O_3_/Mn_2_O_3_(3 : 1), MnFe_2_O_4_ and their precursors. Fig. 2(a) shows that the precursor of Fe_2_O_3_/Mn_2_O_3_(3 : 1) exhibits two morphologies, which are nanoparticles and nanostructured bulk, while the Fe_2_O_3_/Mn_2_O_3_(3 : 1) catalyst exists as sub-sized porous nanosheets-self-assembled globular structure (Fig. 2(b)). The microspheres of Fe_2_O_3_/Mn_2_O_3_(3 : 1) are 3–4 μm in diameter and the pore size is about 30 nm. From the XRD analysis results shown in Fig. 1(a), it can be inferred that the formation of nanopores is due to the release of CO_2_, which comes from MnCO_3_ decomposition during the calcination process. In particular, mesoporous structure is profitable for the rapid transmission of O_2_, fuel and electrolyte, which can accelerate the redox reaction rate and improve electrochemical performance.^28^ Further, the EDS elemental mappings of Fe_2_O_3_/Mn_2_O_3_(3 : 1) (Fig. 2(c)–(f)) were recorded to obtain elemental distribution of Fe, Mn and O in the structure and it could be observed that the three elements are distributed homogeneously. As shown in Fig. 3(a) and (b), MnFe_2_O_4_ catalyst and its precursor have similar hierarchical structures. The nanoparticles are 20–30 nm in diameter and exhibit self-assembled globular shapes with diameters of 300–500 nm. Moreover, the EDS elemental mapping of MnFe_2_O_4_ clearly indicates that the Fe, Mn and O elements are uniformly distributed (Fig. 3(c)–(f)).

**Fig. 4:**
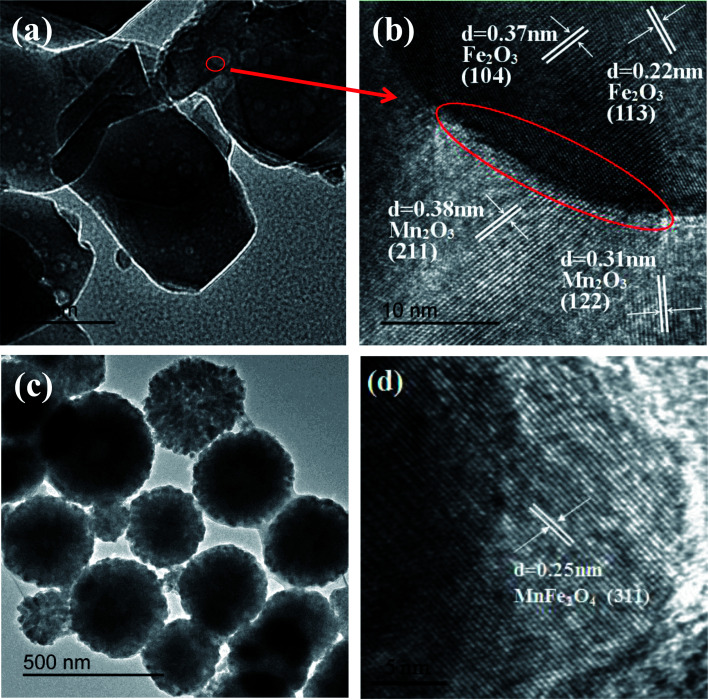
(a) TEM and (b) HRTEM images of Fe_2_O_3_/Mn_2_O_3_(3 : 1); (c) TEM and (d) HRTEM images of MnFe_2_O_4_.

The TEM image of Fe_2_O_3_/Mn_2_O_3_(3 : 1) (Fig. 4(a)) shows that numerous nanoparticles with diameters of 10–30 nm are uniformly dispersed on the substrate. To better characterize the microstructure, a HRTEM image of Fe_2_O_3_/Mn_2_O_3_(3 : 1) was obtained (Fig. 4(b)). The nanoparticle has a clear lattice fringe with *d*-spacing of 0.37 nm and 0.22 nm, corresponding to the Fe_2_O_3_ phase (104) and (113) plane, respectively, while that of the substrate is 0.38 nm and 0.31 nm, corresponding to the (211) and (122) plane of Mn_2_O_3_, respectively. Therefore, Fe_2_O_3_/Mn_2_O_3_(3 : 1) consists of Fe_2_O_3_ and Mn_2_O_3_, which is consistent with the XRD results. As shown in Fig. 4(b), a distinct heterojunction boundary between Fe_2_O_3_ nanoparticles and Mn_2_O_3_ substrate could be detected as shown by the red line. Fig. 4(c) shows that MnFe_2_O_4_ exists as nanospheres with diameters of 300–500 nm. The lattice fringe with *d*-spacing is 0.25 nm, which can be well indexed to the (311) plane of MnFe_2_O_4_ phase (Fig. 4(d)).

**Fig. 5:**
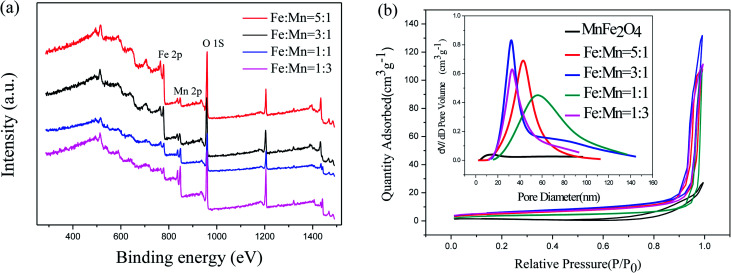
(a) XPS survey spectra of Fe_2_O_3_/Mn_2_O_3_ with different Fe/Mn ratios; (b) nitrogen adsorption–desorption isotherms and pore size distributions of Fe_2_O_3_/Mn_2_O_3_ with different Fe/Mn ratios and MnFe_2_O_4_.

XPS was used to measure the surface chemical composition and confirm the Fe/Mn ratio of the as-prepared Fe_2_O_3_/Mn_2_O_3_ samples. As shown in Fig. 5(a), the common peaks of Fe 2p, Mn 2p and O 1s are present. The element contents are calculated and summarized in Table 1, illustrating that the results of Fe/Mn ratios are approximately equal to the corresponding experimental values. The N_2_ adsorption–desorption technique at 77 K was used to investigate specific surface areas and pore structures of the as-prepared samples. The nitrogen adsorption–desorption curves (Fig. 5(b)) manifest a type IV isothermal line with a delay loop-line in the *P*/*P*_0_ range of 0.9–1.0 for Fe_2_O_3_/Mn_2_O_3_ samples and 0.8–1.0 for MnFe_2_O_4_, indicating porous structures. The BET surface areas are 12.390, 19.889, 21.73 and 18.165 m^2^ g^−1^ for Fe_2_O_3_/Mn_2_O_3_(1 : 1), Fe_2_O_3_/Mn_2_O_3_(1 : 3), Fe_2_O_3_/Mn_2_O_3_(3 : 1) and Fe_2_O_3_/Mn_2_O_3_(5 : 1), while their pore sizes are 55.7, 32.8, 32.8, and 43.7 nm, respectively. MnFe_2_O_4_ illustrates the BET surface area and pore size of 3.05 m^2^ g^−1^ and 14.4 nm, respectively.

**Table 1:** Elemental composition of Fe_2_O_3_/Mn_2_O_3_ with different Fe/Mn ratios

Fe/Mn ratio	Composition			Fe/Mn
	Fe 2p (at%)	Mn 2p (at%)	O 1s (at%)	
	Binding energy			
	708.00 eV	639.00 eV	528.00 eV	
1 : 1	3.77	4.03	92.2	0.94
1 : 3	2.43	7.63	89.94	0.32
3 : 1	9.81	2.84	87.36	3.45
5 : 1	12.10	2.34	85.56	5.17

### 3.2 ORR activity and DMFC performance

**Fig. 6:**
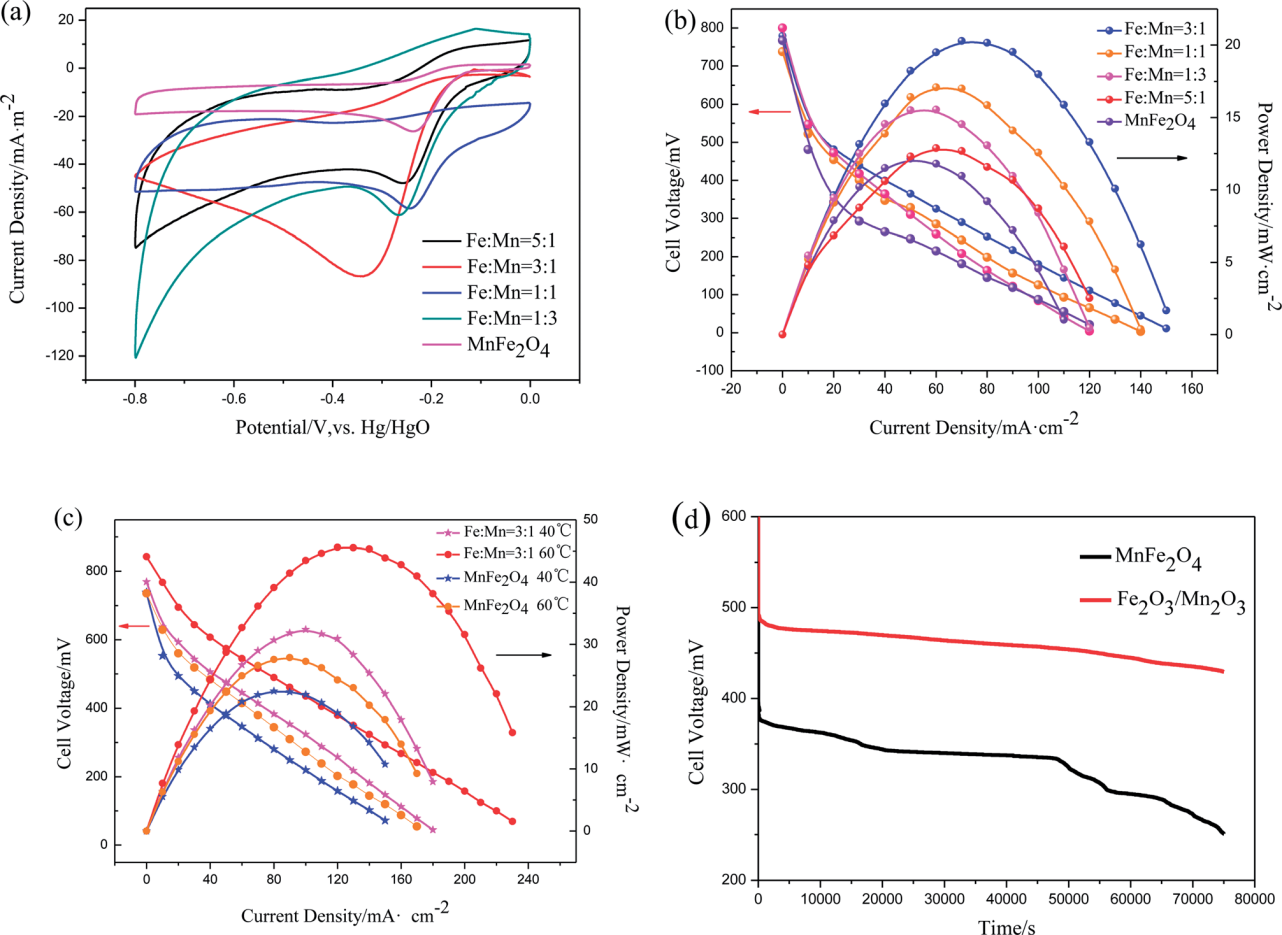
(a) CV curves of Fe_2_O_3_/Mn_2_O_3_ with different Fe/Mn ratios and MnFe_2_O_4_ modified glassy carbon electrodes in O_2_-saturated 1 M KOH solutions under ambient conditions. Scan rate: 50 mV s^−1^. Counter electrode: Pt wire. Reference electrode: Hg/HgO; (b) performance of DMFCs with Fe_2_O_3_/Mn_2_O_3_ with different Fe/Mn ratios and MnFe_2_O_4_ cathode catalysts at room temperature; (c) performance of the Fe_2_O_3_/Mn_2_O_3_(3 : 1)- and MnFe_2_O_4_-based DMFCs at 40 °C and 60 °C; (d) stability tests at the constant current density of 10 mA cm^−2^. Fe_2_O_3_/Mn_2_O_3_(3 : 1) and MnFe_2_O_4_ were employed as the cathode catalysts at room temperature.

CV tests were performed to describe ORR catalytic activity. The current densities were normalized to their corresponding surface area. Capacitance correction was acquired by subtracting the measured current densities under N_2_ from those measured under O_2_ under the same condition. Fig. 6(a) shows the CV curves of Fe_2_O_3_/Mn_2_O_3_ with different Fe/Mn ratios and MnFe_2_O_4_ modified glassy carbon electrodes in O_2_-saturated 1 M KOH solution. Oxygen reduction peaks of these samples are distinct, demonstrating their ORR catalytic activities. Their oxygen reduction peak current densities and corresponding potentials are summarized in Table 2. The oxygen reduction peak current densities are −58.43, −61.21, −86.7 and −47.9 mA m^−2^ for Fe_2_O_3_/Mn_2_O_3_(1 : 1), Fe_2_O_3_/Mn_2_O_3_(1 : 3), Fe_2_O_3_/Mn_2_O_3_(3 : 1) and Fe_2_O_3_/Mn_2_O_3_(5 : 1), respectively, while the corresponding peak potentials are −0.246, −0.267, −0.348 and −0.257 V. Clearly, Fe_2_O_3_/Mn_2_O_3_(3 : 1) has the highest oxygen-reduction peak current density. As compared MnFe_2_O_4_, although the reduction peak potential of Fe_2_O_3_/Mn_2_O_3_(3 : 1) is slightly more negative than that of MnFe_2_O_4_ (−0.237 V), the oxygen-reduction peak current density is much greater than that of MnFe_2_O_4_ (−26.26 mA m^−2^). CV results indicate Fe_2_O_3_/Mn_2_O_3_ exhibits higher ORR activity than MnFe_2_O_4_, which have excluded the effect of their specific surface area, demonstrating Fe_2_O_3_/Mn_2_O_3_ has more active sites probably introduced by heterojunction boundary between Fe_2_O_3_ and Mn_2_O_3_.

**Table 2:** Comparison of the ORR activities and DMFCs performance of the as-prepared samples

Catalysts	Peak current density (mA m^−2^)	Peak potential (V)	BET surface area (m^2^ g^−1^)	*P*_max_ (mW cm^−2^)
Fe_2_O_3_/Mn_2_O_3_(1 : 1)	−58.43	−0.246	12.390	17.09
Fe_2_O_3_/Mn_2_O_3_(1 : 3)	−61.21	−0.267	19.889	15.54
Fe_2_O_3_/Mn_2_O_3_(3 : 1)	−86.7	−0.348	21.73	20.29
Fe_2_O_3_/Mn_2_O_3_(5 : 1)	−47.9	−0.257	18.165	12.88
MnFe_2_O_4_	−26.26	−0.237	3.05	12.15

The polarization and power density curves of Fe_2_O_3_/Mn_2_O_3_ with different Fe/Mn ratios and MnFe_2_O_4_ used as cathode catalysts in DMFCs are shown in Fig. 6(b). The maximum power densities (*P*_max_) for these catalysts are 17.09, 15.54, 20.29, 12.88 and 12.15 mW cm^−2^ for Fe_2_O_3_/Mn_2_O_3_(1 : 1), Fe_2_O_3_/Mn_2_O_3_(1 : 3), Fe_2_O_3_/Mn_2_O_3_(3 : 1), Fe_2_O_3_/Mn_2_O_3_(5 : 1) and MnFe_2_O_4_, respectively. These data indicate that the Fe_2_O_3_/Mn_2_O_3_-based DMFC is superior to MnFe_2_O_4_-based DMFC. As shown in Table 2, Fe_2_O_3_/Mn_2_O_3_(3 : 1) shows the largest peak current density, BET surface area and *P*_max_, illustrating its superior ORR activity. Therefore, Fe_2_O_3_/Mn_2_O_3_(3 : 1) was assigned as Fe_2_O_3_/Mn_2_O_3_ and used for further studies. Fig. 6(c) shows the temperature effects on the DMFCs' performances. The *P*_max_ of Fe_2_O_3_/Mn_2_O_3_- and MnFe_2_O_4_-based DMFCs are 32.4 and 22.5 mW cm^−2^ at 40 °C and 45.6 and 27.9 mW cm^−2^ at 60 °C, respectively. Table 3 compares the *P*_max_ of DMFCs in the literature. In particular, the Fe_2_O_3_/Mn_2_O_3_-based DMFC achieves the highest *P*_max_ among noble and non-noble metal cathode catalysts of DMFCs.

**Table 3:** Comparison of DMFCs performance

Cathode (catalyst loading/mg cm^−2^)	Anode (catalyst loading/mg cm^−2^)	Solution	Electrolyte	Temperature/°C	Power density/mW cm^−2^
Pt/C(8)^33^	PtRu(5)	KOH	Nafion 211	25	15
Pt(1)^34^	Pt(1)	KOH	PVA–KOH	90	10
Pt(1)^35^	PtRu(2)	KOH	PBI/KOH	90	31
Pt(5)^36^	PtRu(5)	KOH	PVA/FS	60	39
Pt/C(10)^22^	PtRu(6)	KOH	Nafion 211	26	16
Pt black(1)^37^	Pt(0.5)	KOH	Nafion 117	60	15
				90	77
Fe-AApyr(7.4)^38^	PtRu(1)	H_2_SO_4_	Nafion 115	30	6.5
				60	18
				90	35
Fe–N–C(4.5)^39^	PtRu(1)	H_2_SO_4_	Nafion 115	90	48
Fe-ABZIM(3)^40^	PtRu(1)	H_2_SO_4_	Nafion 115	60	17
MnO(4)^41^	PtRu(4)	KOH	Q-PVA/PECH	25	17
MnO_2_/C(4)^42^	PtRu black(4)	KOH	PVA/HAP	25	11
MnO_2_(4)^43^	PtRu(4)	KOH	PVA/HAP	25	11
MnFe_2_O_4_(24) (this work)	PtRu/C(5)	KOH	PFM	20	12
				40	22
				60	28
Fe_2_O_3_/Mn_2_O_3_(3 : 1) (24) (this work)	PtRu/C(5)	KOH	PFM	20	20
				40	32
				60	46

Stability tests were conducted in galvanostatic discharge by monitoring the voltage of Fe_2_O_3_/Mn_2_O_3_- and MnFe_2_O_4_-based DMFCs. As shown in Fig. 6(d), at a constant current of 10 mA cm^−2^ at room temperature, the Fe_2_O_3_/Mn_2_O_3_-based DMFC has much higher cell voltage than MnFe_2_O_4_-based DMFC for 75 000 s. In about 50 000 seconds, the voltage of MnFe_2_O_4_-based DMFC decreases sharply. For the Fe_2_O_3_/Mn_2_O_3_-based DMFC, no distinct attenuation phenomenon is found, indicating that this cell is quite stable.

### 3.3 ORR mechanism of Fe_2_O_3_/Mn_2_O_3_

In conclusion, Fe_2_O_3_/Mn_2_O_3_(3 : 1) exhibits higher ORR activity and superior DMFC performance than MnFe_2_O_4_. The first reason is that Fe_2_O_3_/Mn_2_O_3_(3 : 1) has a much larger specific surface area (21.73 m^2^ g^−1^) than MnFe_2_O_4_ (3.05 m^2^ g^−1^), which plays a key role in enhancing ORR activity, providing numerous active sites and accelerating mass-transfer. It is worth noting that although current densities are normalized to their corresponding surface area in the CV tests, Fe_2_O_3_/Mn_2_O_3_ still demonstrates higher ORR activity than MnFe_2_O_4_.

**Fig. 7:**
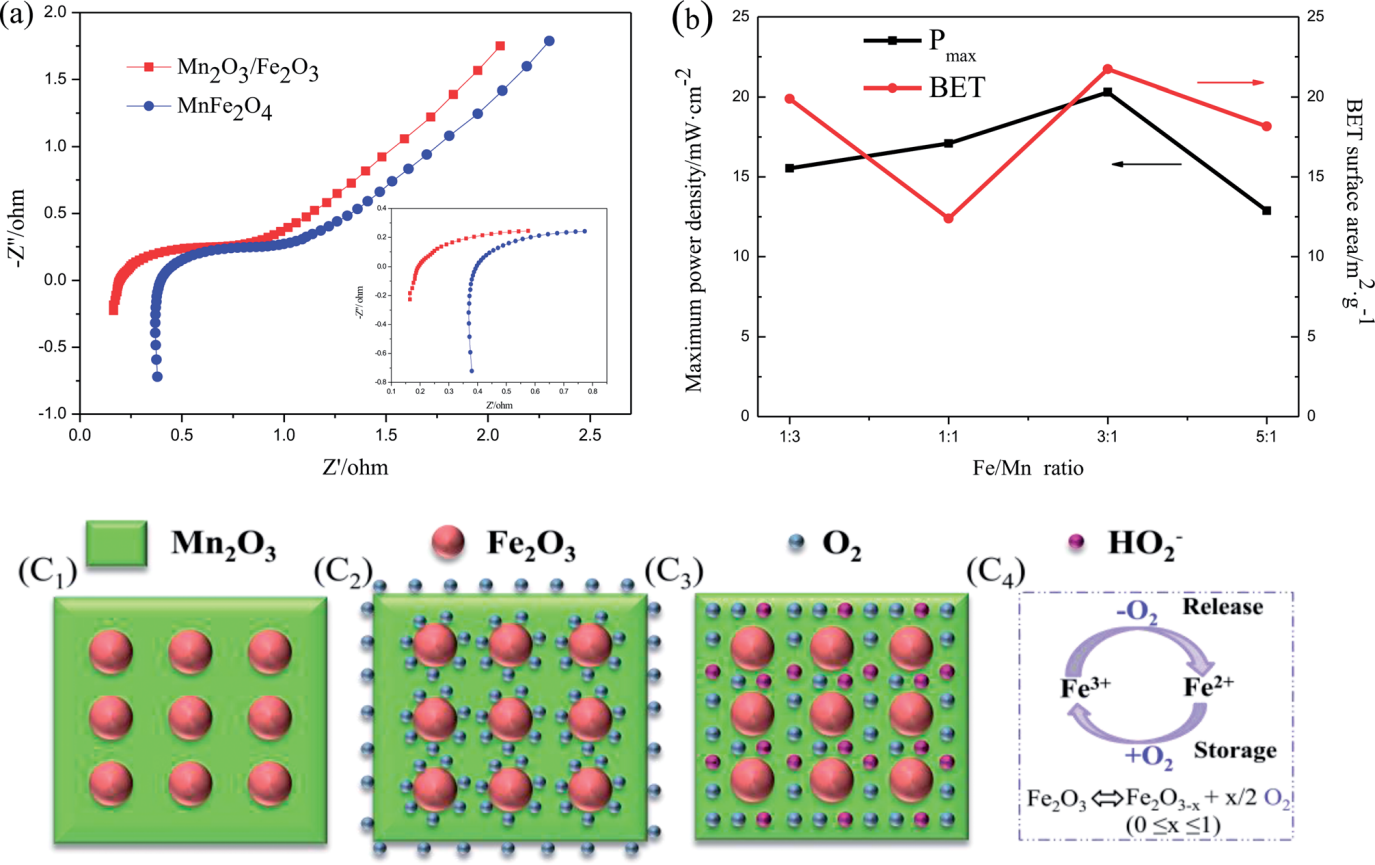
(a) Nyquist plots of the DMFCs with Fe_2_O_3_/Mn_2_O_3_ and MnFe_2_O_4_ cathode catalysts. (Inset: corresponding Nyquist plot in the high-frequency range); (b) *P*_max_ and BET surface area of Fe_2_O_3_/Mn_2_O_3_ with different Fe/Mn ratios; (c) schematic diagrams of ORR mechanism with Fe_2_O_3_/Mn_2_O_3_ as the cathode catalysts; (c_1_) the microstructure of Fe_2_O_3_/Mn_2_O_3_; (c_2_) ORR mechanism under O_2_ surplus; (c_3_) ORR mechanism under O_2_ deficiency; (c_4_) illustration of the Fe^2+^/Fe^3+^ ORR mechanism.

The second reason is due to the existence of the numerous heterojunctions between Fe_2_O_3_ and Mn_2_O_3_, which provides an intensive internal electric field at the interface of the two oxides and increases the catalytic active sites, electron transfer and ORR efficiency.^29,30^ EIS was applied to describe the internal resistance of Fe_2_O_3_/Mn_2_O_3_(3 : 1) and MnFe_2_O_4_. As shown in Fig. 7(a), the Nyquist plots of the Fe_2_O_3_/Mn_2_O_3_(3 : 1)- and MnFe_2_O_4_-based DMFCs exhibit similar trends. The ohmic resistances (*R*_s_) of the Fe_2_O_3_/Mn_2_O_3_(3 : 1)- and MnFe_2_O_4_-based DMFCs are 0.2 Ω cm^−2^ and 0.4 Ω cm^−2^, respectively. *R*_s_ values are the ohmic resistances of the total cell from the anode to cathode, including the solution, electrodes and membrane resistances. These two cells differ only in the cathode catalysts; they have the same solution (4 M KOH and 5 M methanol), membrane and anode. Therefore, it is speculated that the lower resistance of Fe_2_O_3_/Mn_2_O_3_ is owing to the heterojunction providing an intensive internal electric field and increasing the electron transfer. Moreover, the content of heterojunctions between Fe_2_O_3_ nanoparticles and Mn_2_O_3_ matrix is proportional to the number of Fe_2_O_3_ nanoparticles. In other words, with an increase in the Fe/Mn ratio, the density of heterojunctions gradually increases. As shown in Fig. 7(b), on increasing the quantity of heterojunctions, *P*_max_ is gradually improved. However, when the Fe/Mn ratio reaches 5 : 1, *P*_max_ decreases sharply because numerous Fe_2_O_3_ nanoparticles wrap in the Mn_2_O_3_ matrix, impeding the Mn_2_O_3_ catalytic sites from contacting with O_2_ and electrolyte. In addition, Fe_2_O_3_/Mn_2_O_3_(1 : 1) has smaller specific surface area but higher power density than Fe_2_O_3_/Mn_2_O_3_(1 : 3), indicating the ORR activity follows the order of Fe/Mn ratio instead of its specific surface area.

The third reason is the synergistic effect between Fe_2_O_3_ and Mn_2_O_3_ in Fe_2_O_3_/Mn_2_O_3_. The Fe_2_O_3_ particles not only enhance the dispersity of Mn_2_O_3_, but also increase the O_2_ storage capability. Fe_2_O_3_ is an n-type semiconductor with a large number of oxygen vacancies. Many reports suggest that Fe_2_O_3_ has the outstanding ability of reversibly exchanging O_2_ through the transformation of Fe^3+^ and Fe^2+^.^31,32^ As shown in Fig. 7(c), Fe_2_O_3_ acts as an O_2_-storage and release site owing to the Fe^3+^/Fe^2+^ redox couple. It can store O_2_ in O_2_-surplus condition and release it under oxygen deficiency condition. When O_2_ concentration is sufficient, Fe_2_O_3_ captures the surrounding O_2_ molecules on its surface by oxidation reaction from Fe^2+^ to Fe^3+^ as shown in Fig. 7(c_2_). Moreover, when O_2_ is insufficient, such as at high current density, the adsorbed O_2_ on the Fe_2_O_3_ surface can release and obtain electrons, thus forming HO_2_^−^, which rapidly transfers to adjacent catalytic sites of the Mn_2_O_3_ matrix *via* reduction reaction from Fe^3+^ to Fe^2+^ as illustrated in Fig. 7(c_3_). The rapid supply of excess O_2_ and HO_2_^−^ can increase O_2_ transfer and ORR efficiency in Fe_2_O_3_/Mn_2_O_3_. Therefore, the synergistic coupling between Fe_2_O_3_ and Mn_2_O_3_ greatly promotes its superior ORR ability over MnFe_2_O_4_. However, excess Fe_2_O_3_ will reduce ORR ability owing to its poorer intrinsic ORR activity compared to Mn_2_O_3_. Above all, the larger specific surface area, large number of heterojunction interfaces, and excellent synergistic effect of Fe_2_O_3_ and Mn_2_O_3_ play key roles in the enhanced ORR activity of Fe_2_O_3_/Mn_2_O_3_.

## 4. Conclusions

(1) Fe_2_O_3_/Mn_2_O_3_ and MnFe_2_O_4_ were synthesized *via* a facile template-free solvothermal method. Fe_2_O_3_/Mn_2_O_3_ exists as sub-size porous nanosheets-self-assembled globular structures. The microspheres are 3–4 μm in diameter and the pore size is about 30 nm. The formation of nanopores is due to the release of CO_2_, which comes from MnCO_3_ decomposition during the calcination process. The TEM and HRTEM images show Fe_2_O_3_ nanoparticles uniformly dispersed on the Mn_2_O_3_ substrate and a distinct heterojunction boundary between Fe_2_O_3_ nanoparticles and Mn_2_O_3_ substrate. MnFe_2_O_4_ has a hierarchical structure, in which the nanoparticles are 20–30 nm in diameter and the self-assembled globular shapes have diameters of 300–500 nm.

(2) CV and DMFC performance tests show that Fe_2_O_3_/Mn_2_O_3_(3 : 1) exhibits higher ORR activity than Fe_2_O_3_/Mn_2_O_3_(1 : 1), Fe_2_O_3_/Mn_2_O_3_(1 : 3), Fe_2_O_3_/Mn_2_O_3_(5 : 1) and MnFe_2_O_4_. The *P*_max_ of Fe_2_O_3_/Mn_2_O_3_(3 : 1)-based DMFCs are 32.4 and 45.6 mW cm^−2^ at 40 and 60 °C, respectively. The results indicated that the as-prepared Fe_2_O_3_/Mn_2_O_3_ catalysts achieved the highest *P*_max_ among noble and non-noble metal cathode catalysts of DMFCs.

(3) The much superior catalytic performance of Fe_2_O_3_/Mn_2_O_3_ is due to its larger surface area, the existence of numerous heterojunction interfaces and the synergistic effect between Fe_2_O_3_ and Mn_2_O_3_, which can provide numerous catalytic active sites, accelerate mass transfer, and increase ORR efficiency. It is worth noting that Fe_2_O_3_ acts as an O_2_-storage and release site owing to the Fe^3+^/Fe^2+^ redox couple. In addition, the synergistic effect between Fe_2_O_3_ and Mn_2_O_3_ greatly promotes its ORR properties.

## Conflicts of interest

There are no conflicts to declare.

## Supplementary Material
